# Early Descriptions of Family Caregivers' Experiences With Unexpected Lucidity

**DOI:** 10.1093/geroni/igab046.178

**Published:** 2021-12-17

**Authors:** Kyungmin Kim, Joan Griffin, Lauren Bangerter, Virginia Biggar, Dawn Finnie, Theresa Frangiosa, Joseph Gaugler, Maria Lapid

**Affiliations:** 1 Seoul National University, Seoul, Seoul-t'ukpyolsi, Republic of Korea; 2 Mayo Clinic College of Medicine, Rochester, Minnesota, United States; 3 UnitedHealth Group, Minnetonka, Minnesota, United States; 4 UsAgainstAlzheimer's, Washington, District of Columbia, United States; 5 Mayo Clinic, Rocheste, Minnesota, United States; 6 University of Minnesota, Minneapolis, Minnesota, United States; 7 Mayo Clinic, Rochester, Minnesota, United States

## Abstract

To develop an operational definition of and typologies for episodes of lucidity (EL), we conducted a cross-sectional study of former/current family caregivers from UsAgainstAlzheimer’s A_LIST (N = 538). More than 60% of caregivers (n = 294, 62%) reported witnessing EL with their care recipient over the course of their dementia. Most episodes happened in late stages of dementia (71%). Only 10% happened within 7 days before death. The majority of episodes (71%) lasted <30 minutes. About half the episodes were characterized by uncharacteristic speech and communication. Caregivers perceived these experiences positively (M = 4.10; range = 1–5), but also expressed desire to know why/when EL occurs and how to best respond to it. Data will be used to refine definitions and typologies, and then a prospective, demographically diverse survey will be administered to family caregivers to assess predictors of EL, linking EL to caregiver well-being and bereavement response.

